# *Chengia laxispicata* gen. et sp. nov., a new ephedroid plant from the Early Cretaceous Yixian Formation of western Liaoning, Northeast China: evolutionary, taxonomic, and biogeographic implications

**DOI:** 10.1186/1471-2148-13-72

**Published:** 2013-03-27

**Authors:** Yong Yang, Longbiao Lin, Qi Wang

**Affiliations:** 1 State Key Laboratory of Systematic and Evolutionary Botany, Institute of Botany, Chinese Academy of Sciences, 20 Nanxincun, Xiangshan, Beijing 100093, China; 2 China Railway Group Limited, 69 Fuxing Road, Beijing, 100039, China

**Keywords:** Biogeography, *Chengia*, Early Cretaceous, Ephedraceae, Ephedroid, Evolution, Female cone, Gnetales, Jehol biota, Reduction and sterilization hypothesis, Yixian Formation

## Abstract

**Background:**

The extant Gnetales include three monotypic families, namely, Ephedraceae (*Ephedra*), Gnetaceae (*Gnetum*), and Welwitschiaceae (*Welwitschia*), all of which possess compound female cones that comprise a main axis and 1 to multiple pairs/whorls of bracts subtending a female reproductive unit or having lower pairs/whorls of bracts sterile. However, the evolutionary origin of such a reproductive architecture in Gnetales is controversial in the light of the competing anthophyte versus gnetifer hypotheses of seed plant relationships. Hence, macrofossils demonstrating the structure of compound female cones of the Gnetales should be important to decipher the early evolution of the order.

**Results:**

A new ephedroid plant *Chengia laxispicata* gen. et sp. nov. is described from the Early Cretaceous Yixian Formation of western Liaoning, Northeast China. The fossil represents a part of a leafy shooting system with reproductive organs attached. The main shoot bears internodes and swollen nodes, from which lateral branches arise oppositely. Reproductive organs consist of female spikes terminal to twigs or axillary to linear leaves. Spikes are loosely arranged, having prominent nodes and internodes. Bracts of the spikes are decussately opposite and comprise 4—8 pairs of bracts. Each bract subtends an ellipsoid seed. Seeds are sessile, with a thin outer envelope and a distal micropylar tube.

**Conclusions:**

*Chengia laxispicata* gen. et sp. nov. provides a missing link between archetypal fertile organs in the crown lineage of the Gnetales and compound female cones of the extant Ephedraceae. Combined with a wealth of *Ephedra* and ephedroid macrofossils from the Early Cretaceous, we propose a reduction and sterilization hypothesis that the female cone of the extant Ephedraceae may have stemmed from archetypal fertile organs in the crown lineage of the Gnetales. These have undergone sequentially intermediate links similar to female cones of Cretaceous *Siphonospermum, Chengia*, and *Liaoxia* by reduction and sterilization of the lower fertile bracts, shortenings of internodes and peduncles as well as loss of reproductive units in all inferior bracts. The basal family Ephedraceae including *Ephedra* of the extant Gnetales was demonstrated to have considerable diversity by the Early Cretaceous, so an emended familial diagnosis is given here. The Jehol Biota in Northeast China and adjacent areas contains a plethora of well-preserved macrofossils of *Ephedra* and ephedroids that show different evolutionary stages including primitive and derived characters of Ephedraceae, so Northeast China and adjacent areas may represent either the centre of origination or one of the centres for early diversification of the family.

## Background

**Figure 1:**
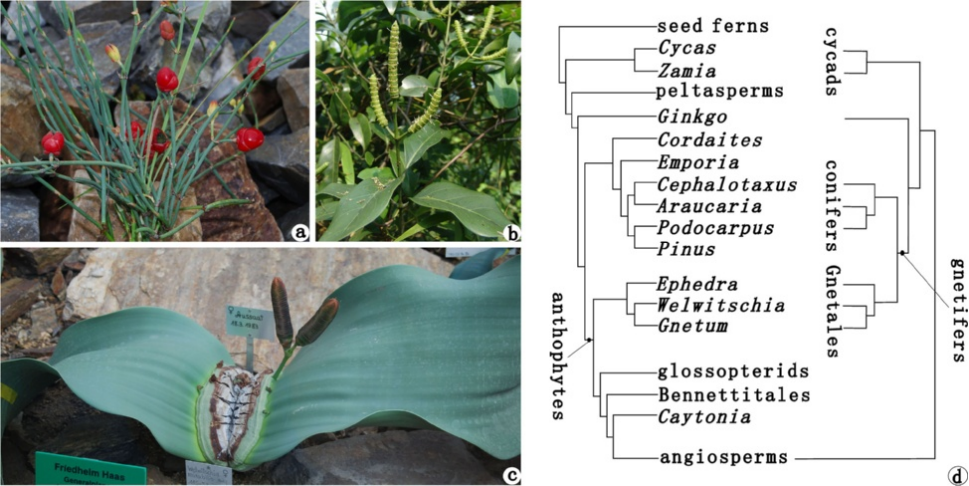
**Three extant genera and phylogenetic relationships of the Gnetales.** (**a**) *Ephedra minuta* Florin photographed at Chayu, Xizang (Tibet), China. (**b**) *Gnetum parvifolium* (Warb.) C.Y. Cheng ex Chun photographed at Fairy Lake Botanic Garden, Shenzhen, Guangdong Province, China. (**c**) *Welwitschia mirabilis* Hook. f. photographed at Dahlem Botanic Garden, Berlin, Germany. (**d**) Simplified phylogenetic trees of the Gnetales within seed plants (after [14]). Left: Gnetales are placed at the base of an anthophyte clade in the morphology tree. Right: Gnetales are embedded within conifers forming a gnetifer clade in the molecular tree.

The extant Gnetales, which are often classified into three monotypic families, namely, Ephedraceae Dumort., Gnetaceae Lindl., and Welwitschiaceae Markgr., have attracted extensive attention because of their enormous potential for understanding the origin of angiosperms and the phylogenetic relationships of seed plants due to the bisexual reproductive units that occasionally occur in *Ephedra* L. [1-4], vessels and broad leaves with reticulate venation present in *Gnetum* L. [5,6], ovules with multiple envelopes [7-10], and double fertilization [11-13]. This order now includes three relict genera *Ephedra*, *Gnetum*, and *Welwitschia* Hook. f., with a mutually exclusive geographic distribution [6,7,9]. Each of three genera possesses compound female cones that comprise a main axis and 1 to multiple pairs of opposite and decussate bracts or 1 to multiple whorls of ternately arranged bracts which usually subtend a female reproductive unit (Figure 1a–c). To date, there are primarily two competing hypotheses on the early evolution and phylogeny of the Gnetales [14-18] (Figure 1d). One hypothesis places the Gnetales into an anthophyte clade [19] using morphological data, which implies that the compound female cones of the Gnetales might be closely related to either flowers of angiosperms or fertile organs of Bennettitales, Erdtmanithecales, and other anthophytes [20-26] (Figure 1d, left). The other hypothesis nests the Gnetales within or closely related to conifers using molecular data, forming either gnepines or gnetifers or gnecups [27-31] (Figure 1d, right), which infers that the compound female cones of the Gnetales and coniferophytes may be homologous. Therefore, the evolutionary origin and phylogenetic relationships of the Gnetales within seed plants remain controversial, and macrofossils especially regarding compound female cones should be important to decipher the early evolution of the order and their relationship with other seed plants.

The extant Ephedraceae (*Ephedra*) usually occupy a basal position in phylogenetic trees of the Gnetales (Figure 1d) while *Gnetum* and *Welwitschia* are more derived and are more closely related to each other than either is to *Ephedra*[14,17-23,27-33]. *Ephedra* has scale-like, linear and 2 (—3) parallel-veined leaves which are often opposite and decussate at nodes and connate at the base into a sheath (e.g., *E. equisetina* Bunge, *E. distachya* L., *E. sinica* Stapf, *E. pedunculata* Engelm. ex S. Wats., and *E. chilensis* C. Presl), and ternate phyllotaxis is also not rare (e.g., *E. intermedia* Schrenk ex C. A. Mey., *E. przewalskii* Stapf, and *E. ochreata* Miers) [4,6,7,9] (Figure 1a). In contrast, *Welwitschia* bears two opposite, enormous strap-like leaves with numerous parallel primary veins (and cross veins with apically oriented chevrons in cotyledons) [6,7,9] (Figure 1c), while *Gnetum* possesses petiolate, broad-laminar and reticulate-veined leaves that are arranged in opposite and decussate manner in most cases [6,7,9] (Figure 1b), and ternate phyllotaxis is also present (Yong Yang, unpublished observations). Bracts and reproductive units of female cones of *Ephedra* and *Welwitschia* are opposite and decussate in most cases while those of *Gnetum* are usually whorled at nodes with the fusion of bracts into cupules and occasionally they are spirally arranged [34-36]. Both *Gnetum* and *Welwitschia* bear female cones with many whorls of fertile bracts while living *Ephedra* normally has only the uppermost whorl/pair of bracts fertile [37-39], rarely the inferior bracts subtending female reproductive units [8,40], or hermaphroditic cones with the lower whorls of bracts bearing male reproductive units but the uppermost whorl possessing female reproductive units [41,42]. In addition, ovules of the Gnetales have 1—2 outer envelopes and the inner integument upwardly extended into a characteristic micropylar tube. Therefore, well-preserved female reproductive organs of the Gnetales may be easily recognized in the fossil record, and macrofossils of the basal family Ephedraceae are especially important in understanding the origin and phylogenetic relationships of the extant Gnetales.

So far, a variety of pre-Cretaceous macrofossils have been attributed to or compared with extant Gnetales (e.g., *Palaeognetaleana auspicia* Z.Q. Wang [43], *Dechellyia gormanii* Ash [9,44], *Dinophyton spinosus* Ash [9,45,46], *Nataligma dutoitii* J.M. Anderson et H.M. Anderson [9], *Sanmiguelia lewisii* Cornet [47,48], *Archaestrobilus cupulanthus* Cornet [49], *Ephedrites sinensis* Wu et al. and *Ephedrites exhibens* Wu et al. [50,51]), but their putative relationships to the Gnetales are not unequivocal due to lacking of synapomorphies recognized from the clade (e.g., compound female cones, whorled, opposite and decussate phyllotaxis in leaves, bracts, and bracteoles) and other detail (e.g., polyplicate pollen *in situ*) as Crane [52] previously suggested. Hence, the extant genera *Ephedra*, *Gnetum*, and *Welwitschia*, together with two Cretaceous genera *Drewria* Crane et Upchurch [53] and *Eoantha* Krassilov [54,55], were considered to form a crown group of the Gnetales [56] while those pre-Cretaceous genera were assigned to either stem-gnetaleans [9] or seed plants with uncertain affinities [48,52]. Recently, a wealth of additional Early Cretaceous macrofossils and mesofossils assignable to the crown-gnetaleans have been widely described from South Europe, Northeast China, Mongolia, North America, South America, and Australia [57], suggesting that three families Ephedraceae, Gnetaceae, and Welwitschiaceae of the extant Gnetales existed and diversified during at least the Early Cretaceous. Of all, macrofossils closely related to *Welwitschia* and *Gnetum* are very rare [58-60], but macrofossils and mesofossils (i.e., seeds) assignable to Ephedraceae or even to the extant *Ephedra* have been extensively reported [61-82]. These members of the Ephedraceae show a very high morphological diversity in both reproductive and vegetative organs across the world except for Africa and Antarctica. According to characteristics of female reproductive organs, Cretaceous ephedroid macrofossils can be classified into three groups, namely: (1) those with female cones bearing 1(—2?) pairs of bracts with only the uppermost pair fertile, e.g., *Ephedra carnosa* Yang et Wang [61], *E. archaerhytidosperma* Yang et al. [62], *E. hongtaoi* Wang et Zheng [63] and *E. verticillata* Cladera et al. [64] as well as *Alloephedra xingxuei* Tao et Yang [57,65,66] and two species of *Gurvanella* Krassilov 1982 [67-69] (= *Chaoyangia* Duan 1998 [70-73], non *Chaoyangia* Hou et Zhang 1993 [83] for an Early Cretaceous bird fossil also from Chaoyang District of Liaoning Province, Northeast China; for nomenclatural discussions see [57,67,69,84]); (2) those with female cones possessing multiple whorls of fertile bracts each subtending a female reproductive unit, e.g., numerous species of *Liaoxia* Cao et Wu 2006 [74] (= *Ephedrites* Göppert et Berendt 1845 [75], non *Ephedrites* Saporta 1891[67,76]); and (3) those that have ovules surrounded by envelopes lacking supporting bracts and directly attached on the peduncles, e.g., *Siphonospermum simplex* Rydin et Friis [77]. Therefore, the basal gnetalean family Ephedraceae, including the extant genus *Ephedra*, was demonstrated to have considerable diversity by the Early Cretaceous.

In this paper, we study a new ephedroid macrofossil from the Early Cretaceous Yixian Formation of western Liaoning Province, Northeast China, and name it *Chengia laxispicata* gen. et sp. nov.. Our new plant bears a comparatively complete, leafy shooting system with loose female spikes that allow us to discuss the evolution of female cones of the Ephedraceae. We also consider the taxonomic and biogeographic implications of our findings on the basis of abundant ephedroid macrofossils from the Early Cretaceous Jehol Biota of Northeast China and adjacent areas.

## Results

### Description of the specimens

**Figure 2:**
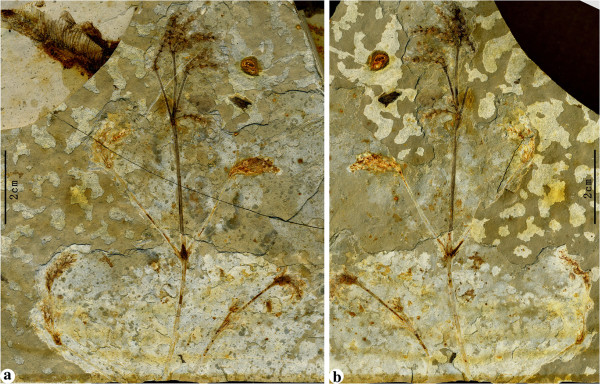
**The general morphology of *Chengia laxispicata *gen. et sp. nov..** Holotype: PE 2012041619A, B. A gathering of part (**a**) and counterpart (**b**) specimens. Note that there is a piece of *Lycoptera* fish fossil exposed on the bedding plane below this plant-bearing bed.

**Figure 3:**
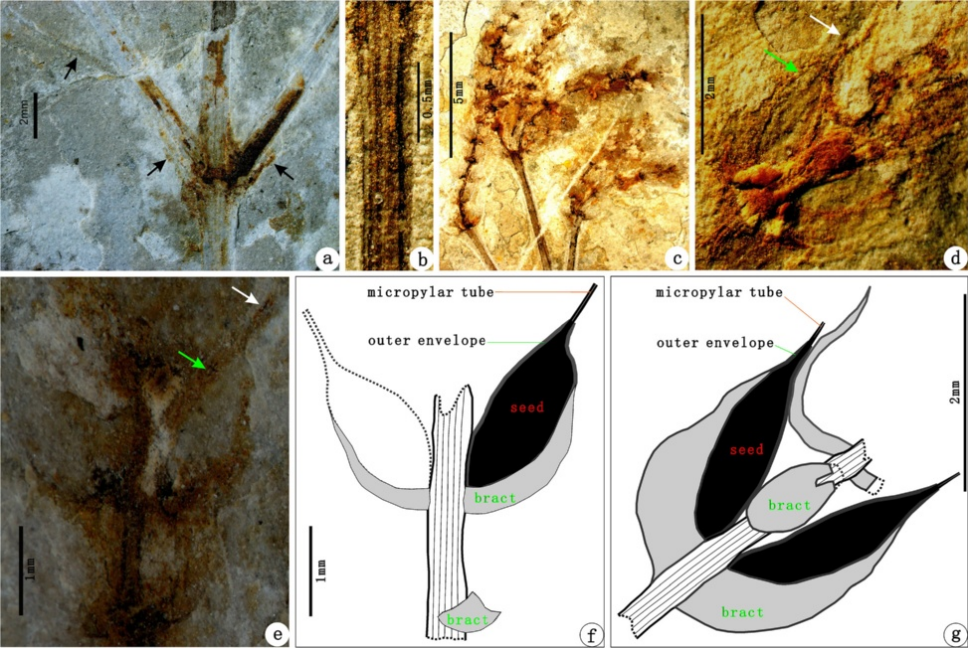
**Morphological detail of *Chengia laxispicata *gen. et sp. nov..** (**a**) A node showing the opposite branching pattern and subtending elongate-linear leaf (arrows). (**b**) An internode showing the fine longitudinal striations. (**c**) Terminal female spikes with the loosely arranged bracts and prominent internodes. (**d–e**) The morphology of female reproductive units. Green arrows refer to an enveloped seed axillary to a bract. White arrows refer to a thin, hollow micropylar tube. (**f**–**g**) The camera lucida drawing of partial spikes in (**e**) and (**d**), showing the decussate and opposite fertile bracts.

**Figure 4:**
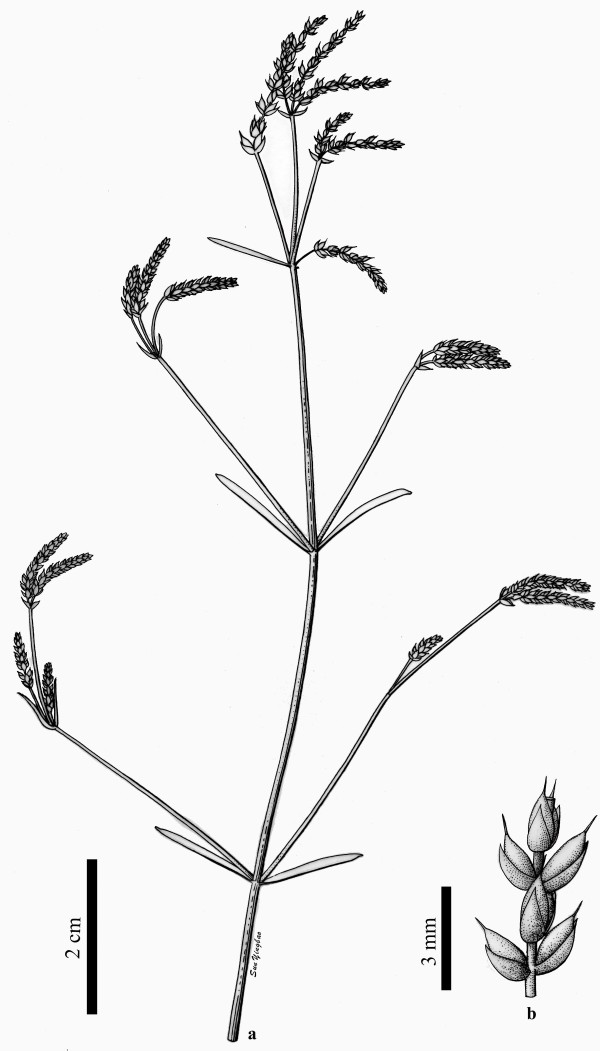
**A reconstruction of *Chengia laxispicata *gen. et sp. nov..** (**a**) The general morphology of *Chengia laxispicata*. (**b**) The morphology of a partial female reproductive spike.

The macrofossils presented here based on information from the part and counterpart of a single well-preserved and articulated specimen (PE 2012041619A, B) that represents part of a leafy shooting system with reproductive organs attached (Figure 2a—b). The shooting system represents the distal part of the plant and is preserved at least 10.2 cm long, and the shoot is ca. (0.21—) 0.53—1.06 (—1.2) mm wide. The main shoot is slightly curved, with nodes and internodes. Nodes are swollen, from which the lateral branches arise oppositely (Figure 2a—b, Figure 3a). Internodes are 2—3.8 cm long or more, taper toward the distal region and have numerous fine, longitudinal striations, in which ridges and furrows are discernible (Figure 3b). The main shoot branches at least 4 times. Lateral branches are stretched upwards at 15—70° and do not ramify or dichasially ramify once to twice. Leaves, which subtend lateral branches, are linear, about 1.2 cm long, but the venation is indiscernible (Figure 3a). Foliar structures at the base of spikes are specialized into involucres which are linear, about 1.3 mm long, approximately perpendicular to the shoot at the proximal part and slightly curved inward at the distal part (Figure 3c). Spikes are terminal to twigs or are axillary to leaves, 1—2.6 cm long and 2—4 mm wide, and mature acropetally (Figure 2a—b). Spikes are loosely arranged at the distal end of the main shoot, but more compact on the basal lateral branches (Figure 2a—b). Those loosely arranged spikes are noticeable in having prominent nodes and internodes (Figure 3c). Nodes are swollen, and each node gives rise to two opposite and spreading bracts. Internodes are approximately 1—2 mm long, acropetally becoming successively shorter and shorter (Figure 3c). Bracts of spikes are decussately opposite and comprise 4—8 pairs that are linear in lateral view (Figure 3d—g). Each bract subtends an ellipsoid seed, about 1.85—2.23 mm long and 1.05—1.16 mm wide. Seeds are sessile, with a thin outer envelope and a distal, hollow micropylar tube, about 0.34—0.75 mm long (Figure 3d—g). Spikes of this plant are all female, which are oriented toward one side of the shooting system and appear to have been flexible rather than rigid. A reconstruction of this plant is given here (Figure 4a—b).

## Discussion

### Identity and affinity

The fossil specimens studied here obviously possess opposite organographic features, including opposite lateral branches, leaves as well as decussately opposite bracts and enveloped seeds, all of which conform to the diagnostic and synapomorphic features of the Gnetales [6,7,9,19,32,52,85,86]. Hence this plant can be readily classified into the Gnetales, and its linear leaves and decussately opposite bracts that comprise female spikes resemble those of the ephedroids. However, the decussately opposite bracts and loose female spikes with clear nodes and internodes are noticeably different from all known ephedroid fossils [52,57] and extant *Ephedra* that bears female cones usually having 2—13 pairs/whorls of cone bracts but with only the uppermost pairs/whorls of bracts being fertile [4,6,7,9]. Therefore, the fossils presented here do not conform to the generic circumscription of *Ephedra* and require a new name for which we institute *Chengia laxispicata* gen. et sp. nov. and place it within the ephedroid Gnetales (See **Conclusion—*Systematics***).

### The evolution of female cones in Ephedraceae

Previous morphological, anatomical, ontogenetic, and molecular studies supported a reduction hypothesis that female cones of the extant Ephedraceae may have stemmed from a loosely arranged, multi-axial, reproductive organs homologous to the Late Palaeozoic Cordaitales and Permian—Triassic coniferophytes [4,6,8,15,17,35,36,38-40],[87-94]. With shortening of internodes of the reproductive multi-axial shoot system, both the primary and secondary shoots of Ephedraceae, to some extent, may have experienced a series of structural reductions and finally given rise to compact female cones in extant *Ephedra*[4,8,87,89]. Hence, female cones similar to laxly arranged reproductive shooting systems would represent more primitive organizations than those resembling to compact reduced female cones of the extant Ephedraceae. Our new fossil *Chengia laxispicata* gen. et sp. nov. bearing loose female spikes appears to be a missing link to the extant Ephedraceae with compact cones.

Early Cretaceous macrofossils have provided diverse characteristics of female reproductive structures. Ovulate cones of *Ephedra carnosa*[61]*, E. archaeorhytidosperma*[62], *E. hongtaoi*[63], *Gurvanella dictyoptera* Krassilov and *G. exquisita* Sun et al. [67-69,80] from the Early Cretaceous Jehol Biota of Northeast China and adjacent areas bear either 1(—2?) pairs or 1 whorl of bracts enclosing 1—3 seeds, effectively demonstrating the same pattern as modern *Ephedra* that possesses only the uppermost one pair/whorl of fertile bracts. *Siphonospermum simplex* has linear leaves, opposite phyllotaxis, and ovules with exposed micropylar tubes and surrounded by envelopes, which are attached directly on the peduncles, so these ephedroid features may be plesiomorphic in the Gnetales [77]. Female spikes of *Liaoxia* have multiple whorls of fertile bracts each subtending a female reproductive unit (or seed) (referring to *Liaoxia robusta* Rydin et al. [74]), which provide palaeobotanical evidence for the previous morphological—evolutionary interpretation that the archetypal female cone of Ephedraceae may be compound, with multiple-whorled fertile bracts.

Our new ephedroid fossil-genus *Chengia* markedly differs from both extant and fossil species of *Ephedra* by the female cone bearing multiple (4—8) pairs of fertile bracts. It is different from *Siphonospermum* by the female reproductive unit possessing subtending bracts. *Chengia* also differs from *Liaoxia* in having loosely arranged female cones. The female spikes of *Chengia* have evident nodes and internodes showing similarity to the supposed laxly arranged reproductive shoot system of ancient ancestors, so such female cones in Ephedraceae are more primitive than those of *Liaoxia* and other ephedroid fossils (e.g., *Alloephedra, Gurvanella*, and *Ephedra*).

**Figure 5:**
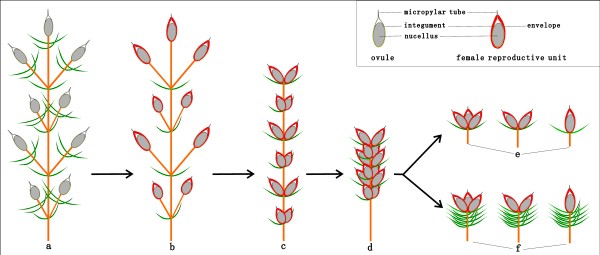
**A reduction and sterilization hypothesis on the evolutionary pathways of female cones of Ephedraceae.** (**a**) A hypothesized archetypal fertile organs of the crown lineage of the Gnetales. (**b**) Female reproductive units directly terminal to peduncles and bearing no supporting bracts like ephedroid macrofossil *Siphonospermum simplex* Rydin et Friis [77]. (**c**) A loosely arranged female cone having multiple pairs of fertile bracts like ephedroid macrofossil *Chengia laxispicata* gen. et sp. nov. presented herein (Figure 2a–b). (**d**) A compact female cone bearing multiple whorls of fertile bracts like ephedroid macrofossil *Liaoxia robusta* Rydin et al. [74]**.** (**e**) Reduced tri-ovulate, bi-ovulate, and uni-ovulate female cones bearing only one whorl/pair of fertile bracts like ephedroid macrofossils *Ephedra carnosa* Yang et Wang [61], *E. archaeorhytidosperma* Yang et al. [62], *E. hongtaoi* Wang et Zheng [63], and *Gurvanella exquisita* Sun et al. and *G. dictyoptera* Krassilov [67-69]. (**f**) Reduced and compact female cones of modern *Ephedra* species bearing only the terminal bract or the uppermost pair/whorl of bracts fertile and the inferior bracts sterile [4,6,7,9].

*Chengia* together with other macrofossils from the Early Cretaceous Yixian Formation of Northeast China give instrumental clues to the morphological transformation of cone bracts in Ephedraceae. According to current phylogenetic trees of the Gnetales [14,17,18] (Figure 1d), the ancestral form of the order is far more likely to bear compound female cones regardless of the anthophyte hypothesis or gnetifer hypothesis. Here, we propose a reduction and sterilization hypothesis that the female cone of *Ephedra* is derived from that of *Siphonospermum*, *Chengia*, and *Liaoxia* by a series of changes that include reduction and sterilization of lower fertile bracts, shortenings of internodes and peduncles, loss of reproductive units in all inferior bracts, and retains only the uppermost pair/whorl of bracts fertile (Figure 5).

## Conclusion

This ephedroid plant *Chengia laxispicata* gen. et sp. nov. described from the Early Cretaceous Yixian Formation of Northeast China provides a missing link between archetypal fertile organs of the crown lineage of the Gnetales and compound female cones of the extant Ephedraceae. Based upon a plethora of *Ephedra* and ephedroid macrofossils from the Early Cretaceous, we propose a reduction and sterilization hypothesis that the female cone of the extant Ephedraceae may have stemmed from archetypal fertile organs of the crown lineage of the Gnetales, which have undergone sequentially intermediate links similar to female cones of Cretaceous *Siphonospermum, Chengia*, and *Liaoxia* by a series of transformations that include reduction and sterilization of the lower fertile bracts, shortenings of internodes and peduncles, loss of reproductive units in all inferior bracts. The basal family Ephedraceae including *Ephedra* of the extant Gnetales was demonstrated to have considerable diversity by the Early Cretaceous, so an emended familial diagnosis is given here. The Jehol Biota in Northeast China and adjacent areas contains a plethora of well-preserved macrofossils of *Ephedra* and ephedroids that show different evolutionary stages including primitive and derived characters of Ephedraceae, so Northeast China and adjacent areas may represent either the centre of origination for the family or its centre of early diversification.

### Systematics

Gnetales Luerss. 1879

Ephedraceae Dumort. 1829, emend.

**Type:***Ephedra* L. 1753, Sp. Pl. 1040.

**Familial diagnosis emended** (based on previous work    [7,95-99] and present study)

Dioecious (rarely monoecious) shrubs, sub-shrubs, small tree, climbers, or sometimes perennial herbs. Shoots are profusely and dichasially branched and have many nodes; nodes are usually swollen, sometimes branches are whorled at nodes due to extremely reduction of internodes or alternate on account of suppression of the opposite branch; internodes of twigs possess many fine striations. Leaves are opposite and decussate or ternately whorled at nodes, linear and free to basally connate into a sheath but apices acute and triangular. Leaves are parallel veined, with 2 (—3) veins. Reproductive organs are usually unisexual but hermaphroditic cones occasionally occur. Male cones are terminal to twigs, or pedunculate, or sessile and axillary to leaves, or clustered at nodes, bear multiple pairs/whorls of bracts each of which enclose an axillary male reproductive unit. The male reproductive unit is a shortened shoot and usually consists of a pair of bracteoles enclosing 1 microsporangiophore. The microsporangiophore is fused or distally furcated, and terminated by 2—8 synangia. The synangium is sessile to pedicellate, consists of 2—3 microsporangia, the latter opening by horizontal slits. At maturity, the microsporangiophore is elongated outside the bracteoles and terminated by a few free or fused synangia which produce polyplicate pollen bearing longitudinal ridges and furrows. The female reproductive units (FRUs) or compound female cones are directly terminal to twigs, or pedunculate or sub-sessile or sessile at nodes. The compound female cones if present are trimerous or bimerous, bear 1 to multiple whorls/pairs of leaf-like or specialized bracts, each subtending a female reproductive unit, or only the uppermost whorl/pair of bracts being fertile and the inferior whorls/pairs of bracts becoming sterile. Cone bracts are sometimes modified into dry and membranous, or dry and coriaceous, or fleshy and colourful. Seeds bear an outer envelope and an inner integument which usually extends upward and passes through the opening of the outer envelope, forming a thin and hollow micropylar tube. Micropylar tubes varying in length and shape, 0.2—4 mm long, straight, curved or coiled.

#### Remarks

In 1829, the family Ephedraceae was instituted by the Belgian botanist Barthélemy-Charles Dumortier (1797—1878) [100], who only gave a brief familial diagnosis “ovaire supère stylifère; écailles opposes; ovaire monogyne”. In modern plant taxonomy, the Ephedraceae are usually characterized by features of the sole extant *Ephedra*[7,95-99]. However, abundant ephedroid fossil plants from the Early Cretaceous have increasingly broadened our understanding of the circumscription and character variation of the family, so an emended familial diagnosis is provided here. The key diagnoses (Figure 5) of Ephedraceae include enveloped ovules with extruded micropylar tubes (i.e., female reproductive unit), linear leaves opposite and decussate or ternately whorled and having 2—3 parallel veins. In light of the present study, Ephedraceae *sensu lato* contain *Ephedra* L. with ca. 50 living species, 4 macrofossil-species (i.e., *E. carnosa* Yang et Wang, *E. archaeorhytidosperm* Yang et al., *E. hongtaoi* Wang et Zheng, *E. verticillata* Cladera et al. [61-64]), and 2 seed fossil-species (i.e., *E. portugallica* Rydin et al. and *E. drewriensis* Rydin et al. [82]) as well as ephedroid macrofossils *Alloephedra* Tao et Yang [57,65,66], *Gurvanella* Krassilov [67-69], *Liaoxia* Cao et Wu [74], *Siphonospermum* Rydin et Friis [77], *Leongathia* Krassilov et al. [78], *Amphiephedra* Miki [101], and *Chengia* gen. nov. presented herein, indicating higher generic diversity through time. Early Cretaceous strata of Northeast China contain a plethora of well-preserved macrofossils (that sometimes are comparatively complete individuals) of *Ephedra* and ephedroids that show different evolutionary stages including primitive and derived characters of Ephedraceae, and in this respect Northeast China and adjacent areas that yield the famous Jehol Biota [102-112] might represent either the centre of origination or one of centres for early diversification of the family.

***Chengia laxispicata*** Y.Yang, L.B.Lin et Q.Wang gen. et sp. nov. (Figures 2, 3 and 4).

#### Etymology

The generic name “*Chengia*” is dedicated to the late eminent botanist Cheng Wan-Chun (1904—1983) (Chinese Academy of Forestry, Beijing) who has made enormous contributions to the taxonomy of gymnosperms; the specific epithet “*laxispicata*”, stemming from the Latin “*laxus*” + “*spicatus*”, refers to the loosely arranged female spike which is a conspicuous feature of this fossil-species.

#### Generic and specific diagnosis

Reproductive shoots branch oppositely at swollen nodes. Internodes bear numerous longitudinal striations. Leaves that subtend lateral branches are long and linear. Female spikes are terminal to twigs or are subtended by the leaf, possessing multiple pairs of loosely arranged female reproductive units. Nodes and internodes of female spikes prominent. Female reproductive units consist of decussately opposite seeds and subtending bracts. Seeds enveloped, with distal micropylar tube.

**Description**: (see **Results*—Description of the specimens***).

#### Holotype

PE 2012041619A, B (Figure 2a***—***b) (designated here. Part and counterpart specimens).

#### Type locality

**Figure 6:**
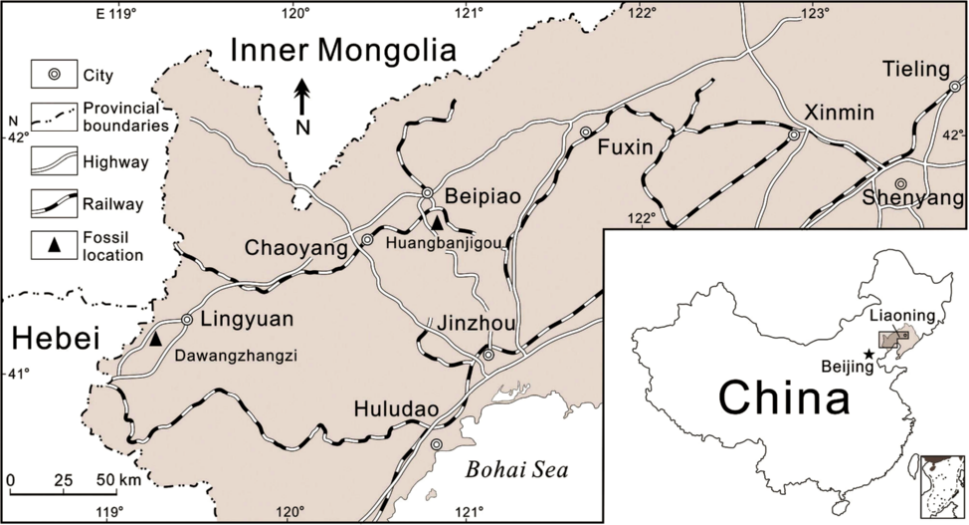
**The geographic positions of ephedroid macrofossil localities in western Liaoning Province, Northeast China.** Left: Dawangzhangzi Village, Lingyuan City—the type locality of *Chengia laxispicata* gen. et sp. nov.. Middle: Huangbanjigou Village, Beipiao City—a previously reported locality abundant in ephedroid macrofossils.

Dawangzhangzi Village, Songzhangzi Town, Lingyuan City, Chaoyang District, Liaoning Province, China (Figure 6, left).

#### Stratigraphic horizon and age

Dawangzhangzi Bed in the middle part of the Yixian Formation or Xinfangzi Bed in the lower part of the Yixian Formation (the Early Aptian—earliest Late Aptian of the Early Cretaceous) (see **Method**s as follows).

#### Repository

Chinese National Herbarium (PE), Institute of Botany, Chinese Academy of Sciences, Beijing, China.

#### Remarks

The plant-bearing beds at Dawangzhangzi Village (Figure 6, left), Lingyuan City, Chaoyang District, Liaoning Province, Northeast China have been known since the 1930s. Fossil fish *Lycoptera jeholensis* Grabau, monocotyledonous plants *Potamogeton jeholensis* Yabe et Endô, *Potamogeton* ? sp., gymnosperms *Schizolepis jeholensis* Yabe et Endô and *Czekanowskia rigida* Heer were first reported from this Early Cretaceous locality [113,114]. Subsequently, *Potamogeton jeholensis* was reclassified as another angiosperm *Ranunculus jeholensis* (Yabe et Endô) Miki [101] or an ephedroid plant *Ephedrites chenii* (Cao et Wu) Guo et X.W. Wu [67,76] (correctly cited as *Ephedrites cheniae* Guo et X.W. Wu [57,75]), which is synonymous with *Liaoxia cheniae* (Guo et X. W. Wu) Cao et S. Q. Wu [74]. Moreover, Miki [101] described a new ephedroid plant *Amphiephedra rhamnoides* Miki from this locality, which is different from our new plant *Chengia laxispicata* gen. et sp. nov. in bearing “verticillate, scaly leaves on the lateral branches that look more like short shoots” (translated from the original description in Japanese by Atsushi Yabe, 2012, personal communication).

Adjacent to Dawangzhangzi Village of Lingyuan City, an ephedroid plant *Ephedrites? elegans* Sun et Zheng [67] was described from the Jianshangou Bed in the lower part of the Yixian Formation of Huangbanjigou Village (Figure 6, middle), Beipiao City, Chaoyang District, Liaoning Province, Northeast China. *Ephedrites? elegans* (specimens examined: PB 19175A, B) also bears loosely arranged spikes with clear nodes and internodes, but it differs from our new plant *Chengia laxispicata* gen. et sp. nov. in having possibly 3—5 bracts per whorl in spikes (see original authors’ description) [67]. Another superficially similar specimen was also reported from the Early Cretaceous *Lycoptera* beds of Manlaj, eastern Gobi in Mongolia, which Krassilov called “*Potamogeton*-like spike” bearing 2 or 3 nutlets per node (see original author’s description) [68].

## Methods

The specimens used in this study were collected from Dawangzhangzi Village (Figure 6, left), Lingyuan City, Liaoning Province, Northeast China and occur as part and counterpart on a slab of light grey to yellowish, finely laminated siltstone (Figure 2a–b). The plant fossils are preserved as compressions/impressions only with little organic material remaining, and a piece of *Lycoptera* fish fossil is also present on the same slab. The stratum yielding the present specimens belongs to the informally named “*Lycoptera* Beds” (i.e., Xinfangzi Bed), which corresponds to the “Jianshangou Bed” in the lower part of the Yixian Formation of Huangbanjigou Village, Beipiao City, Liaoning Province, Northeast China [67] (Figure 6). However, some palaeontologists [104-107] designated the statum as the Dawangzhangzi Bed, which corresponds to the middle part of the Yixian Formation, overlying the Jianshangou Bed. On the basis of radiometric dating, the Jianshangou and Dawangzhangzi Beds of the middle-lower Yixian Formation are given a geological age ranging from ca. 125 to 120 Ma [104-112], corresponding to the Early Aptian—earliest Late Aptian of the Early Cretaceous in the newest Geologic Time Scale (GTS 2012) [115].

The fossils (Figures 2 and 3) were photographed with digital cameras (Nikon D700 and Panasonic DMC—FZ30) and under a microscope (Nikon Eclipse E600). Previously published ephedroid macrofossils from the lower part of the Yixian Formation of Huangbanjigou Village (Figure 6, middle), Beipiao City, Liaoning Province, Northeast China were examined at the Institute of Botany (CAS), Beijing (specimens prefixed PE), and Nanjing Institute of Geology and Palaeontology (CAS), Nanjing (specimens prefixed PB). The modern gnetalean plants (Figure 1a–c) were photographed respectively at Chayu (Tibet, China), Shenzhen Fairy Lake Botanic Garden (Guangdong, China), and Dahlem Botanic Garden (Berlin, Germany). Simplified phylogenetic trees (Figure 1d) of the Gnetales within seed plants were adapted from the literature [14]. The illustrations (Figure 4a–b) were drawn using a pointed pen and black inks [116]. The illustrations (Figures 5 and 6) were drawn using CorelDraw 10.0 programme (Chinese edition, Tianlong Corporation, Beijing).

To comply with requirements of *the International Code of Nomenclature for algae, fungi, and plants (Melbourne Code)*[117], we have deposited paper copies of this article in libraries at the Institute of Botany, Chinese Academy of Sciences, Beijing; Peking University, Beijing; the Institute of Vertebrate Paleontology and Paleoanthropology, Chinese Academy of Sciences, Beijing; Nanjing Institute of Geology and Palaeontology, Chinese Academy of Sciences, Nanjing; Kunming Institute of Botany, Chinese Academy of Sciences, Kunming; Sun Yat-sen University, Guangzhou; Lanzhou University, Lanzhou; Jilin University, Changchun; Shenyang Normal University, Shenyang; National Museum of Natural History, Smithsonian Institution, Washington, D.C.; Indiana University, Bloomington; University of California Museum of Paleontology, Berkeley; Florida Museum of Natural History, the University of Florida, Gainesville; Peabody Museum of Natural History, Yale University, New Haven; Ohio University, Athens; the University of Kansas, Lawrence; the University of Birmingham, Birmingham; National Museum Wales, Cardiff, UK; the Swedish Museum of Natural History, Stockholm; Geological Institute, ETH, Zürich; Charles University, Prague; Institute of Evolution, University of Haifa, Israel; the Staatliche Museum für Naturkunde, Stuttgart; Hungarian Natural History Museum, Budapest; Chuo University, Tokyo; National Museum of Nature and Science, Tsukuba, Japan; National Institute of Carpology, Moscow; Far Eastern Geological Institute, Far Eastern Biological and Soil Institute, the Academy of Sciences of the USSR, Vladivostok; The University of Adelaide, Adelaide, Australia.

## Competing interests

The authors declare that they have no competing interests.

## Authors’ contributions

YY and QW conducted photographing specimens, data analyses, and evolutionary interpretations and wrote the manuscript. LBL collected the type specimens and participated in discussions. QW arranged the figures and formatted the text. All authors read and approved the final manuscript.
